# Amelioration of Neuropathic Pain and Attenuation of Neuroinflammation Responses by Tetrahydropalmatine Through the p38MAPK/NF-κB/iNOS Signaling Pathways in Animal and Cellular Models

**DOI:** 10.1007/s10753-021-01593-x

**Published:** 2021-11-10

**Authors:** Cheng Hu, Menglin He, Meijuan Chen, Qian Xu, Sha Li, Yaomei Cui, Xizi Qiu, Weiqian Tian

**Affiliations:** 1 Department of Anesthesiology, Affiliated Hospital of Nanjing University of Chinese Medicine, Jiangsu Province Hospital of Chinese Medicine, Nanjing, Jiangsu 210029 China; 2 School of Medicine & Holistic Integrative Medicine, Nanjing University of Chinese Medicine, Nanjing, China

**Keywords:** Neuropathic pain, Tetrahydropalmatine (THP), Neuroinflammation, Spared nerve injury (SNI).

## Abstract

Neuropathic pain (NP) treatment remains a challenge because the pathomechanism is not yet fully understood. Because of low treatment efficacy, there is an important unmet need in neuropathic pain patients, and the development of a more effective pharmacotherapy is urgently required. Neuroinflammation induced by oxidative stress-mediated activation of nuclear factor-kappa B (NF-κB) plays an important role in NP. In this study, we aimed to investigate the protective properties of tetrahydropalmatine (THP) on a spared nerve injury (SNI) model of neuropathic pain in mice in in vivo and also in in vitro experiments. THP decreased mechanical hyperalgesia and cold allodynia compared with the SNI group. A microarray was applied to analyze differentially expressed of mRNA among different groups, and THP noticeably changed the expression of MAPK-related proteins compared with the SNI groups. H&E staining showed that the THP changed the inflammation after the spared nerve injury, with decreased NO expression in the THP group as compared to the SNI group. In addition, SNI-induced pain was reversed by intraperitoneal administration of THP, and further results indicated that THP suppressed inducible nitric oxide synthase (iNOS, pro-nociceptive mediators), phosphorylated MAPKs, and p65 in the dorsal root ganglions and sciatic nerve, while the serum levels of the pro-inflammatory cytokines IL-1β were significantly higher in the SNI group as compared to the THP group. To identify the molecular mechanism of the antineuropathic activity of THP, sodium nitroprusside (SNP)-induced neuro-2a (N2a) cells, LPS-induced BV2 cells, and LTA-induced astrocytes were further investigated in signaling pathways. In vitro experiments indicated that THP suppressed the expression of IL-1β, iNOS, phosphorylated MAPKs, and p65, which were assayed using western blotting, and immunofluorescence.

## INTRODUCTION

Neuropathic pain (NP) is one type of pain caused by a lesion or disease of the somatosensory system and can severely affects the quality of life of patients [1]. In the past few decades, NP has become a universal issue in healthcare and affects a wide range of people around the world. At present, the commonly used clinical treatment methods include local nerve block, sympathetic block, physiotherapy, opioid anesthetics, anti-infective agents, antidepressants, and/or anxiolytics. These drugs are not only addictive, but they also have certain unwanted side effects [2, 3], and pain management remains a challenge with their continued use. In addition, the related molecular mechanism of NP is not clear. In order to develop a new strategy for treating NP, it is necessary to more completely understand the mechanism behind it [4]. Current studies have shown that the various biological activities of tetrahydropalmatine (THP) are the inhibition of calcium overload and anti-inflammatory and antiarrhythmic action, while few studies have been conducted to explore its role in spared nerve injury (SNI)-induced neuropathic pain. The aim of this study is to verify the mechanism used by THP to alleviate SNI-induced neuropathic pain and neuroinflammation.

Studies have shown that nitric oxide (NO) is a pivotal nociceptive mediator that plays an important role in chronic pain conditions at the peripheral and central levels [5]. NO is one of the 10 smallest molecules found in nature. It is an unstable nitrogen free radical whose predominant functions are that of a messenger through cGMP. In mammals, NO is synthesized by the enzyme nitric oxide synthase (NOS) in different types of cells when L-arginine is converted to citrulline. There are three isoforms of NOS; (i) neuronal (nNOS, NOS1) and (ii) endothelial (eNOS, NOS3) are constitutive calcium-dependent forms of the enzyme that regulate neural and vascular function, and (iii) iNOS (NOS2) is calcium-independent and inducible [6]. Nitric oxide synthases (NOSs) are a family of isoforms responsible for the synthesis of the potent dilator NO. Inflammation results in the expression of inducible NOS (iNOS) and produces large amounts of NO, which is involved in physiological functions such as neurotransmission and immune response facilitation. In pathological conditions, iNOS is regarded as a harmful enzyme and is has been proposed that it contributes to many diseases, including those of the cardiovascular system and nervous system [7].

Previous studies have shown that various inflammatory stimuli lead to phosphorylation and activation of p38 mitogen-activated protein kinase (MAPK) [8]. The activated MAPK contributes to the activation of nuclear factor-kappa B (NF-κB) via NF-κB translocation. The activated NF-κB promotes production of nuclear factor-kappa B (TNF)-α and interleukin (IL)-1β and activates the downstream signaling pathways that lead to the development and maintenance of pain [9]. NP, which is associated with local neuroinflammation of the nervous system, can lead to disability. NP is mediated by the mechanism of neuroinflammation that affects the nervous system and is controlled by the inflammatory response of the initial injury. Pro-inflammatory cytokines IL-1β can stimulate the secretion of traditional inflammatory mediators, such as iNOS [10].

NF-κB is a multipotent transcriptional regulator that plays an important role in cell survival, immunity, and inflammation. NF-κB is activated when cells undergo oxidative stress, following phosphorylation; NF-κB enters the nuclei and then upregulates the expression of pro-inflammatory cytokines such as TNF-α, IL-1β, and IL-6, which has been well demonstrated to play an important role in the establishment and maintenance of the spinal cord sensitization, resulting in hyperalgesia, which induces NP [11, 12]. As an important mediator of inflammation and immune response, cytokines play a key role in the pathophysiological processes associated with NP. Some studies have shown that after peripheral nerve injury, microglia accumulate in the spinal cord, which then leads to NP. [13].

The aim of this study was to examine the influence of tetrahydropalmatine (THP) on nerve cells activation in a mouse neuropathic pain model (spared nerve injury (SNI) of the sciatic nerve). We analyzed the protein levels of the pronociceptive (IL-1β and iNOS) factors in the dorsal root ganglion (DRG) and sciatic nerve (SN) in the SNI-exposed mice after THP administration. We used neuronal cells, microglial cells, and primary astrocytes cultures to determine how much THP would protect them when they were induced by sodium nitroprusside (SNP), lipopolysaccharide (LPS), and lipoteichoic acid (LTA), respectively. The next step of our work is to determine which signaling pathway is involved after THP changes the pronociceptive factors characteristic of neuropathic pain.

## MATERIALS AND METHODS

### Animals

Male C57BL/6 mice (weighting 23 ± 2 g) were purchased from Qing Longshan for this study. The animals were housed at 23 ± 2℃ and maintained on a 12-h light/dark cycle with food and water ad libitum. All animal experiments were duly approved by the Institute Animal Ethics committee for the use of animal subjects. Animal care and handling procedures were followed according to the guidelines of the International Association for Study of Pain (IASP) for the use of animals in pain research [14]. Behavioral tests were carried out without knowing to which experimental group each mouse belonged.

### Surgical Preparations

The mice were anesthetized with isoflurane and placed on a heating blanket to maintain normothermia while undergoing anesthesia. The lateral surface of the left thigh was shaved using a razor blade, followed by topical application of povidone iodine with a prep pad. A single, small skin incision was made at the mid-thigh level with fine scissors using the femur as a landmark, and a blunt dissection was made using the dull portion of the dissection scissors through the biceps femoris muscle (BFM).The sciatic nerve and its three branches were exposed. For the SNI operation, distal to the trifurcation of the sciatic nerve, the common peroneal and tibial nerves were ligated using nylon suture, and a 2–4-mm piece of each distal nerve stump was removed. The sural nerve was kept intact, and any stretching or contact with the spared sural nerve was avoided. In the sham operation, the aforementioned manipulations of the sciatic nerve and its branches were not performed. Incisions were closed with muscle and skin sutures. Once recovered, the SNI-operated animals resumed normal food intake and growth and displayed regular movements and grooming [15].

### Behavioral Tests

The mechanical pain threshold and cold pain threshold of the injured side of the mice in each group were measured 1 day before surgery, 1 day after surgery, and 3, 5, 7, 10, and 14 days after surgery. In order to observe the analgesic effect of drugs, the THP (40 mg/kg) and an equal volume of vehicle was intraperitoneally injected into mice before behavioral testing. S-Methylisothiourea (SMT) (10 mg/kg) is a preferential inhibitor of inducible nitric oxide synthase (iNOS) for pain, and we added the SMT group as a positive control group [16].

#### Mechanical Withdrawal Threshold (MWT) Testing (von Frey Test)

Mice were placed in plastic cages on an elevated mesh floor and allowed to habituate to the testing environment for 30 min before commencement of the tests. The up and down method was used. Mechanical stimuli-induced pain sensitivity was measured using a series of von Frey filaments. The sural nerve skin territory in the mouse paw was tested with von Frey hair. First, the cilium was vertically punctured into the corresponding skin area of the back paw sole of the mouse and slightly forced until it was bent into an S-shape. According to whether the mouse exhibited a foot retraction reflex, the cilium that administered the next or previous bending force was replaced, lasting for 4–6 s each time, and the test interval was 10 s. A series of reactions of the mice to different folding forces from the cilia were recorded. If the mouse exhibited a rapid foot retraction reflex, licking, or shaking reaction, it was recorded as a positive reaction that was represented by an “X” and was replaced by a cilium with a higher bending force. If there was no reaction, it was regarded as a negative reaction that was represented by an “O” and was replaced by a cilium with a lower bending force. The experiment was terminated if any of the following occurred: (1) five stimulus-responses were measured since the first “X” appeared; (2) no positive or negative response was obtained until the von Frey fiber with the highest or lowest bending force was used. Finally, a series of “O” or “X” sequences was obtained, and the value of PWT was obtained by a certain formula [17].

#### Cold Allodynia Testing

Cold allodynia was measured by the number of withdrawal responses of the hind foot after cold stimulation. The mice were gently restrained in clear plexiglass cages, and their hind paws were maintained in the cold (4–6 °C) for 5 min. Then, the number of leg retractions was measured within 5 min [18]. The number of operated lateral leg contractions or foot additions by the mice was recorded.

### Cell Cultures

N2a cells and BV2 microglia cells were grown in Dulbecco’s modified Eagle’s medium (DMEM) supplemented with 10% fetal bovine serum (FBS), 100 units/ml penicillin, and 100 µg/ml streptomycin (Life Technologies, Frederick, MD, USA). Cells were maintained at 37℃ with 5% CO_2_ in a humidified atmosphere. All test concentrations of compounds exhibited no significant toxicity. The cell viability was determined by Cell Counting Kit-8 (CCK-8) assay. Astrocytes were extracted from fetal mice, and cells were cultured in DMEM/Nutrient Mixture F-12 medium containing 10% FBS and 1% phosphatidylserine (PS). Astrocyte cells were stimulated with lipoteichoic acid (LTA, Sigma, Germany) (1 µg/ml), and similarly, BV2 microglia cells were stimulated with LPS (200 ng/ml), and N2a cells were stimulated with SNP (166 µmol/l) [19–22].

### qRT-PCR Analysis

Total RNA was extracted from tissues using TRIzol reagent (Invitrogen, US) according to the manufacturer’s instructions. mRNA was quantified and then reverse transcribed into cDNA using the 5 × All-In-One RT MasterMix cDNA Synthesis Kit (abm, Canada). EvaGreen 2 × qRT-PCR MasterMix-Low ROX (abm, Canada) was used to quantify fluorescence with specific primers for the mRNA. GAPDH was used as an internal control. All qRT-PCR experiments were performed using the Agilent Technologies Stratagene Mx3000P system (Agilent Technologies, Palo Alto, CA, USA). The data were processed with the 2^−ΔΔCt^ method, and the fold changes were normalized to the expression of the internal controls. The primer sequences are:

(GAPDH-F: CTCTCTGCTCCTGTTCGACAG,

GAPDH-R: GTGTAATCATATTGGAACATGTAG,

iNOS-F: ACTCAGCCAAGCCCTCACCTAC.

iNOS-R: TCCAATCTCTGCCTATCCGTCTCG.

IL-1β-F: TCGCAGCAGCACATCAACAAGAG.

IL-1β-R: AGGTCCACGGGAAAGACACAGG).

### Western Blot Analysis

The tissues or cells were collected, lysed in radioimmunoprecipitation assay (RIPA) buffer, and centrifugated at 12,000 × *g* for 20 min at 4℃. The total protein concentration was measured using a bicinchoninic acid (BCA) protein assay kit. Lysates were mixed with 5 × sodium dodecyl sulfate–polyacrylamide gel electrophoresis (SDS-PAGE) loading buffer and then boiled at 95℃ for 10 min. Samples from different groups containing approximately 20 mg protein were separated by 8–12% SDS-PAGE, and then, the protein in the gel was transferred to polyvinylidene fluoride (PVDF) membranes. Afterward, the PVDF membranes were blocked with 5% (w/v) non-fat milk in phosphate-buffered saline with Tween 20 (PBST) buffer on a shaker for 1.5 h at room temperature. This was followed by incubation with the corresponding primary antibodies p-P38 (1:500, ImmunoWay, USA), iNOS (1:1000, Proteintech, USA), p-P65 (1:500, Beyotime, China), IL-1β (1:500, ImmunoWay, USA), or β-actin (1:2000, ImmunoWay, USA) overnight at 4℃. The membranes were then washed three times for 15 min each time and incubated with horseradish peroxidase (HRP)-conjugated secondary antibodies (1:2500 dilution). Finally, after washing as previously described, antigen–antibody complexes were detected by enhanced chemiluminescence (ECL) chemiluminescent substrate. The levels of protein expression were normalized to the density of β-actin. Fold change in the control group was expressed as 100% for quantification. Images were then used for the final determination of protein expression using Image Lab™ software and were normalized to the loading control.

### Drug Administration

First, 918.6 mg tetrahydropalmatine (THP) (batch number G03D9L76182) (purity > 98%) was added to 24 g ultrapure water. Then, the liquid was stirred while adding 20 ml of 0.05 M sulfuric acid. After stirring for 30 min until the THP completely dissolved, the solution was stirred for an additional 10 min, while the pH was measured. The 0.05 M sulfuric acid was continuously added until the pH of the solution reached approximately 3.0, and the solution was stirred for an additional 5 min. The stirring was stopped, and the weight of the solution was fixed at 60.0 g. S-Methylisothiourea sulfate (MedChemExpress, USA) (SMT) was dissolved in normal saline. SMT for intraperitoneal (i.p) administration was freshly prepared by dispersing in distilled water.

### Enzyme-Linked Immunosorbent Assay (ELISA) Determination of Cytokines in Peripheral Blood

The IL-1β level was measured in serum by an ELISA protocol. For mouse serum preparation: orbital blood was collected, standing for 30 min, and then centrifuged at 3,000 r·min ^−1^ for 15 min. The upper serum was removed from a 1.5-ml Eppendorf tube and stored at − 80℃. IL-1β (ZC-37974, ZCIBIO Technology Co., Ltd.) in the serum of mice was detected by ELISA assay according to the kit instructions. The concentration of IL-1β was calculated by a standard curve.

### Histopathological

On day 14, after the behavioral test, three mice from each group were euthanized with isoflurane, and the DRG (L3 to L6) and sciatic nerve (SN) were dissected. The specimens were immediately fixed in 4% paraformaldehyde overnight and subsequently underwent gradient dehydration to transparency. After dehydration, DRGs and SNs were embedded in paraffin and longitudinally sectioned. The Sects. (10 µm) were mounted on glass slides and deparaffinized in xylene. After the slides were rehydrated by decreasing the concentrations of the ethanol, they were subjected to hematoxylin and eosin (H&E) and toluidine blue staining for proteoglycan for the subsequent histological examination.

### Immunofluorescence

Cells at a density of 1 × 10^5^ cells/well were seeded into 12-well plates. After exposure to the THP treatments, the cells were fixed with 4% paraformaldehyde for 30 min at room temperature. Then, the cells were made transparent with 1% PBST for 10 min at 4℃. After the PBST was aspirated, the cells were blocked with 4% bovine serum albumin (BAS) for 30 min and then incubated with a primary antibody against p-P38 (1:200, ImmunoWay, USA), iNOS (1:200, Proteintech, USA), and GFAP (1:200, CST, USA), in antibody dilution buffer overnight at 4 °C. The next day, the cells were incubated with a fluorochrome-conjugated anti-rabbit secondary antibody (1:200, ZSGB-BIO, China) for 2 h at room temperature in the dark. Subsequently, the cells were stained with 4′,6-diamidino-2-phenylindole (DAPI, 1 μg/ml, ZSGB-BIO, China) for 15 min. Images were obtained under a fluorescence microscope. Quantitative analysis of the immunofluorescent staining was performed using Image J.

### Determination of Nitric Oxide in Serum and Tissue Samples

NO production was analyzed by measuring the accumulated level of its stable metabolite (nitrite). The nitrite levels of samples were measured using the Griess method [23] as an index of NO production. Briefly, samples (50 µl) or sodium nitrite (NaNO2) standard was mixed with 100 µl of Griess reagent (50 µl of 0.1% sulfanilamide in 5% phosphoric acid and 50 µl of 0.1% N-1-naphthylethylenediamine dihydrochloride) and then were incubated at room temperature for 10 min. The absorbance was measured at 550 nm using a microplate reader, and the concentration of NO was calculated using a sodium nitrite standard curve.

### Data Analysis

All statistical analyses were performed using GraphPad Prism software. The data from the study are presented as the mean ± standard deviation of three independent experiments. Multiple groups were compared using a one-way or two-way ANOVA followed by Tukey’s multiple comparison test. *P*-value < 0.05 was considered statistically significant.

## RESULTS

### THP Ameliorates SNI-Induced Pain Behavioral Responses

**Fig. 1:**
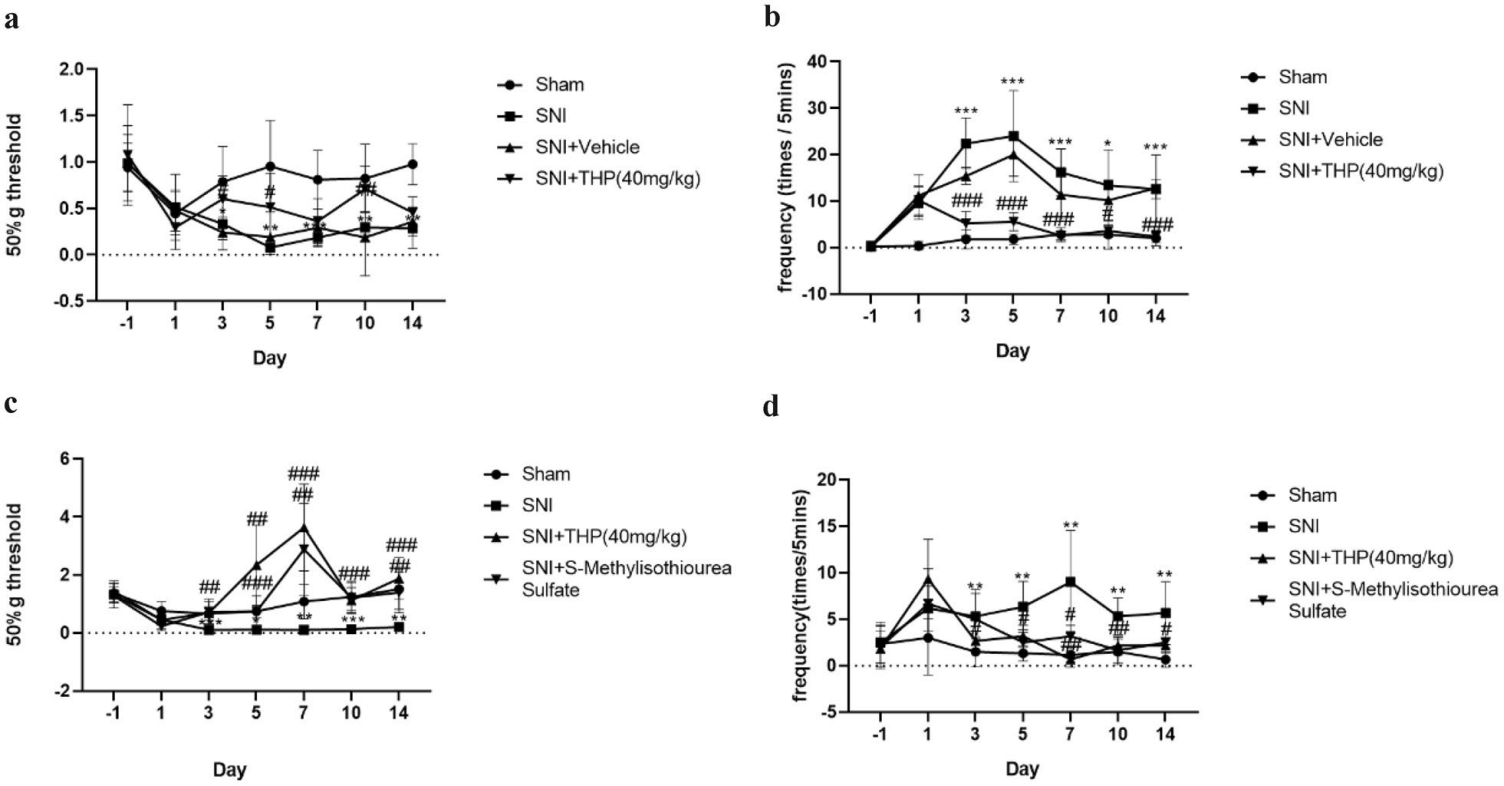
Inhibition of an SNI-induced neuropathic pain model by THP. The mechanical withdrawal threshold (by the Von Frey test) and cold allodynia were measured 1 day before operation, 1 day after surgery, and 3, 5, 7, 10, and 14 days after surgery. The data obtained are expressed as the means ± S.D (*n* = 6 per group); statistical analysis has been carried out using two way ANOVA followed by Tukey’s multiple comparison analysis. (*) *P* < 0.05, (**) *P* < 0.01, and (***) *P* < 0.001 indicate significant differences compared with the Sham group. (#) *P* < 0.05, (##) *P* < 0.01, and (###) *P* < 0.001 indicate significant differences compared with the SNI group.

Mice developed neuropathic pain after SNI. The mice were randomly divided into four groups: Sham, SNI + Vehicle, SNI, and SNI + THP; Sham, SNI, SNI + THP, and SNI + SMT. The mechanical hyperalgesia and cold allodynia behaviors of each group were detected, and there was a decrease in mechanical withdrawal thresholds (MWT) and cold allodynia in the ipsilateral paw from 3 to 14 days after SNI compared to the sham-operated mice. THP was able to relieve these behaviors (Fig. 1a, b). SMT as an inhibitor of inducible nitric oxide synthase (iNOS) used in mice can also alleviate pain behavior (Fig. 1c, d).

### Differentially Expressed mRNA Were Screened Out Through Microarray Hybridization

**Fig. 2:**
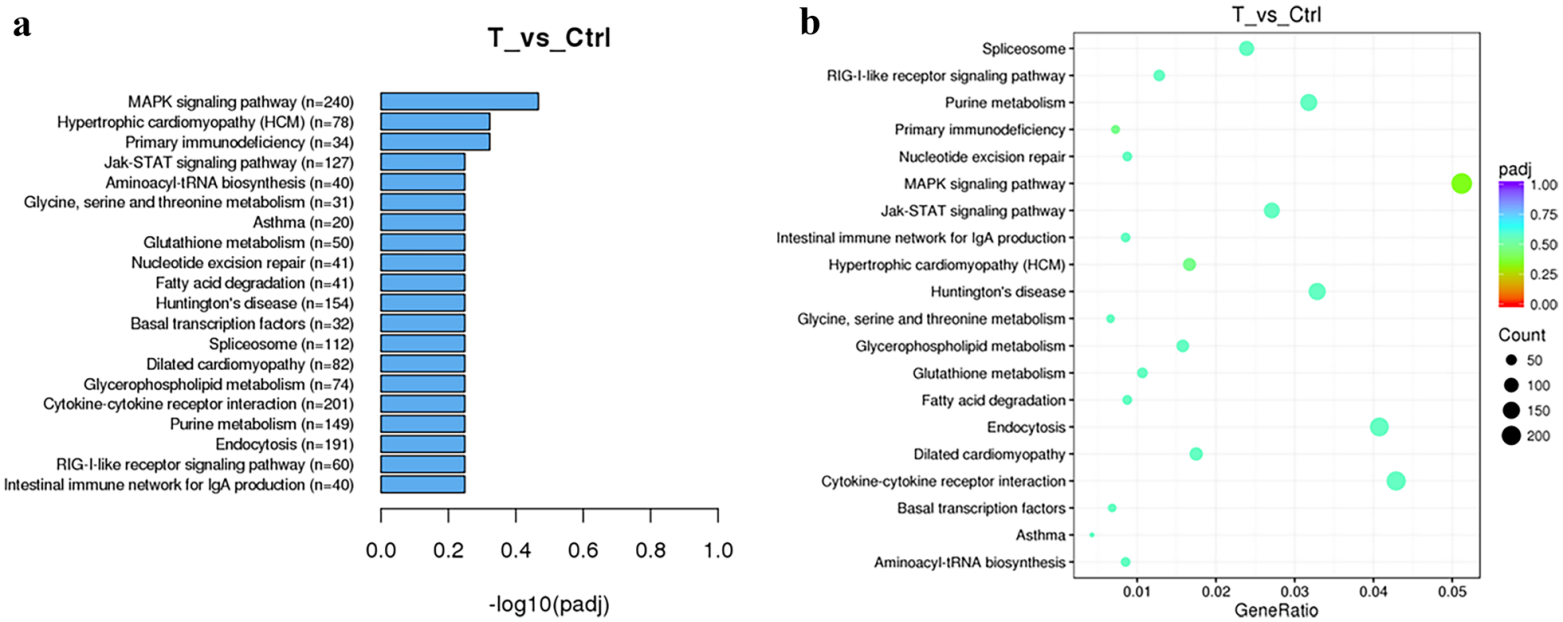
RNA sequencing and analysis by the GO and KEGG databases were performed for the DRG samples. Differentially expressed transcripts (DETs) between the SNI group and the SNI + THP group are listed in the top 20 (*n* = 6 per group). Functional analysis of differentially expressed genes was based on RNA-Seq data.

To investigate the aberrant expression of mRNAs in patients with neuropathic pain, an SNI mouse model was used to simulate human subjects with neuropathic pain, and the mRNA expression profiles of the SNI group and SNI + THP group were analyzed by microarray technique. As shown in Fig. 2a, there was a total of 20 mRNAs that were significantly changed in the SNI group compared with the SNI + THP group. The gene ontology (GO) and Kyoto Encyclopedia of Genes and Genomes (KEGG) analytical data showed that the most enriched mRNA associated with differentially molecular functions was that of MAPK signaling proteins (Fig. 2a, b).

### The Inflammation Levels in SNI Model Mice

**Fig. 3:**
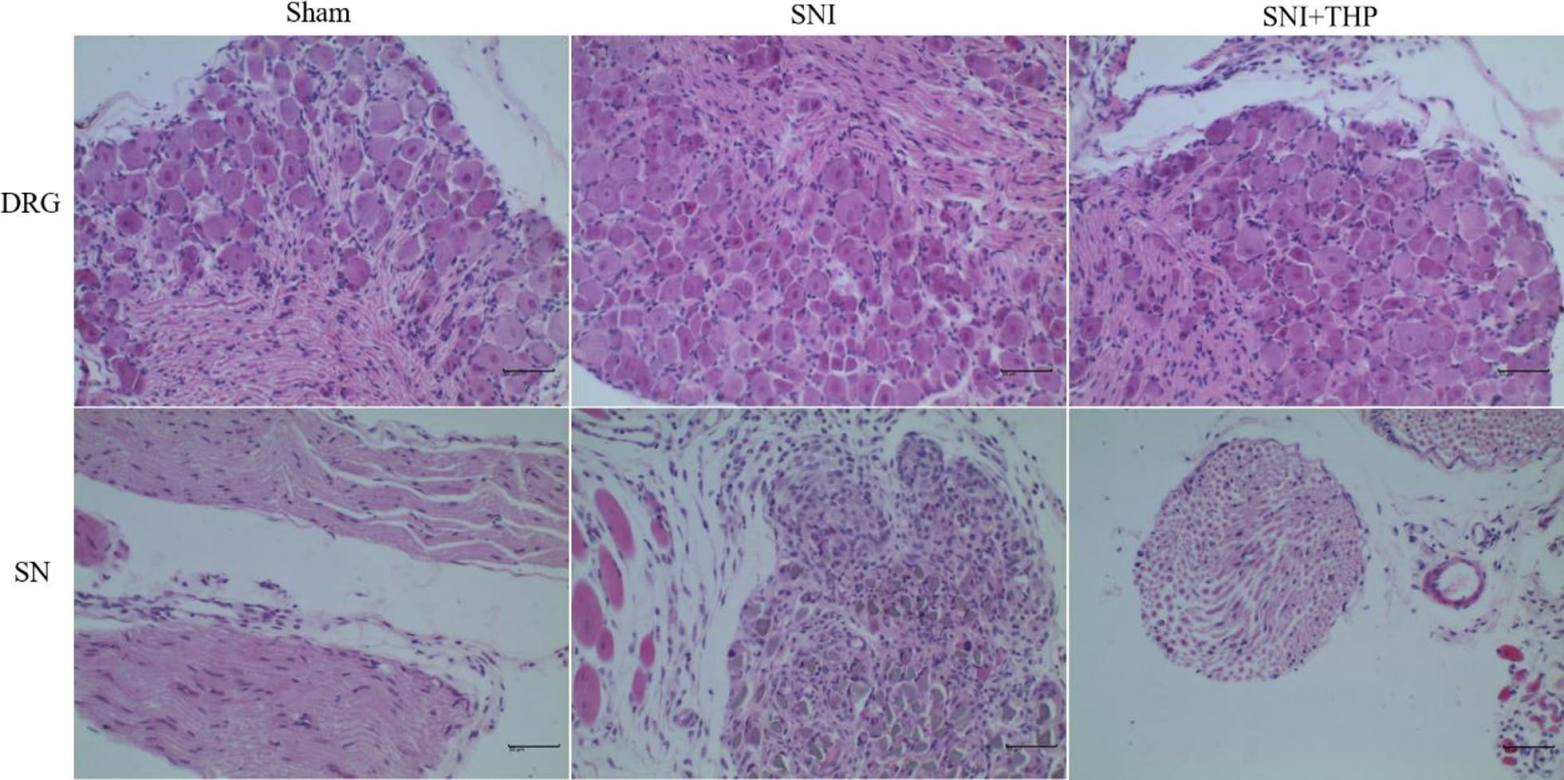
H&E staining of DRGs and SNs was conducted in the in vivo studied groups. H&E staining was conducted for tissue in all studied groups to analyze the level of inflammatory cell infiltration (Scale bar, × 100) (*n* = 3 per group).

More importantly, there was inflammation on the side of the mouse that underwent the operation, while the THP significantly reduced the level of inflammation compared with the SNI group. This effect of THP was further confirmed by examination of hematoxylin and eosin (H&E)-stained DRG and SN sections (Fig. 3). We found that THP reduced inflammation after SNI injury.

### Validation of the Microarray Data by qRT-PCR

**Fig. 4:**
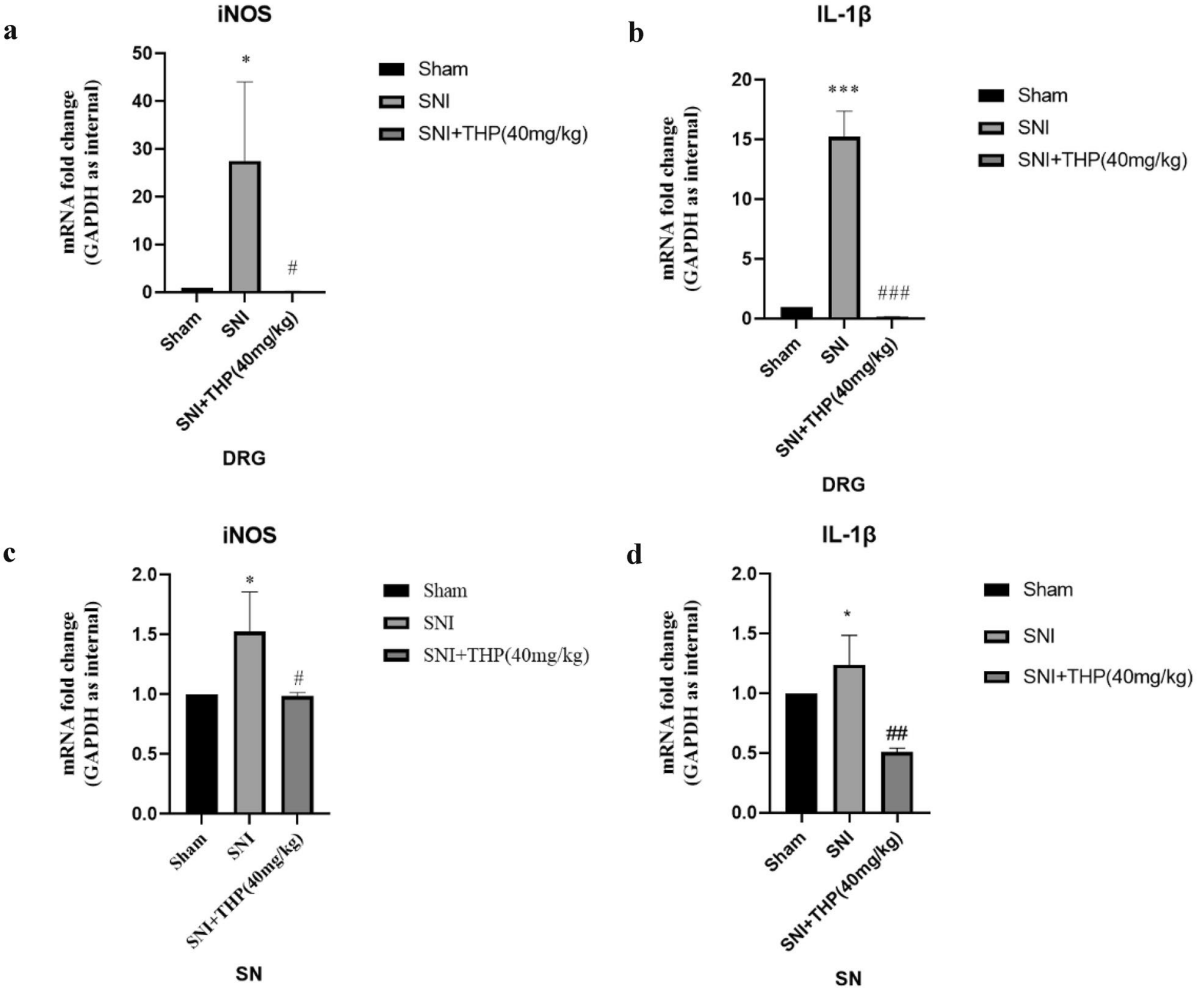
qRT-PCR was used to examine iNOS and IL-1β expression in the DRGs and SNs samples. The expression of iNOS and IL-1β in the SNI group increased compared with the Sham group, while THP reverse this change. Data are expressed as mean ± S.D. (*n* = 3 per group). (*) *P* < 0.05 and (***) *P* < 0.001 indicate significant differences compared with the Sham group. (#) *P* < 0.05, (##) *P* < 0.01, and (###) *P* < 0.001 indicate significant differences compared with the SNI group.

The expression levels of iNOS and IL-1β were measured in DRG and SN tissue samples among three groups (Sham, SNI, and SNI + THP) by qRT-PCR. In the SNI animal model, iNOS and IL-1β mRNA level increased in SNI group compared with the Sham group. THP can reduced the expression of iNOS and IL-1β induced in SNI model. Hence, we next investigated the role of iNOS and IL-1β in regulating neuropathic pain in SNI mice (Fig. 4a–d).

### THP Attenuated the Level of Nitric Oxide and IL-1β in Different Groups

**Fig. 5:**
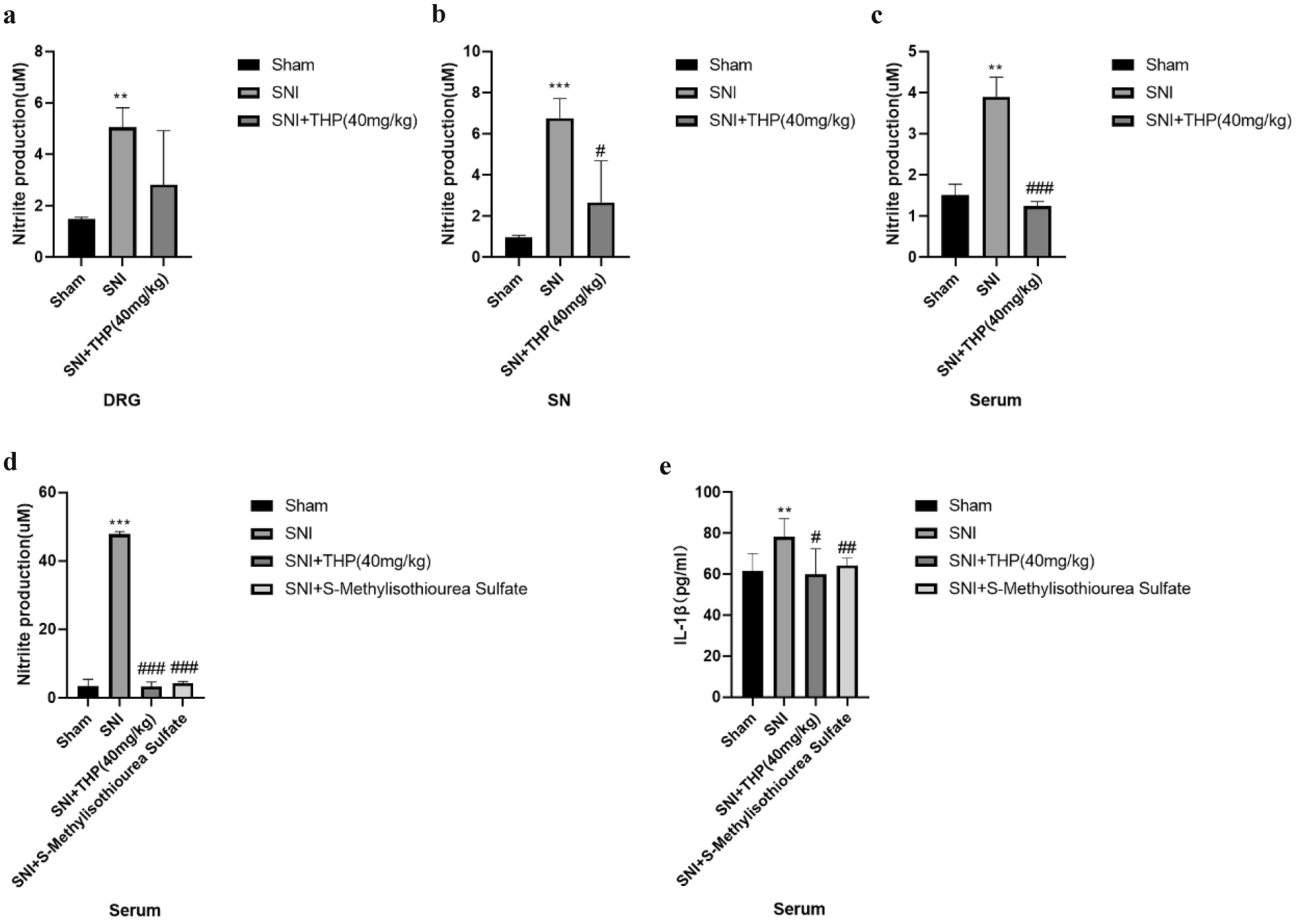
THP attenuated the level of nitric oxide and IL-1β in different groups. Tissue or serum samples nitrite levels were measured using the Griess method. An ELISA kit was used to measure the level of IL-1β in different groups. Data are expressed as mean ± S.D. (*n* = 6 per group). (**) *P* < 0.01 and (***) *P* < 0.001 indicate significant differences compared with the Sham group. (#) *P* < 0.05, (##) *P* < 0.01, and (###) *P* < 0.001 indicate significant differences compared with the SNI group.

SNs, DRGs, and serum samples were separately collected from different groups mice to measure the NO base of nitrite levels, while the serum levels of IL-1β were determined using an ELISA kit for the different groups. The results showed that THP decreased the expression of NO in the SNI-induced pain model, and the expression of IL-1β in serum was decreased in the SNI + THP and SNI + SMT groups compared with the SNI group (Fig. 5a–e).

### THP Beneficially Reduces Proteins in Different Groups

**Fig. 6:**
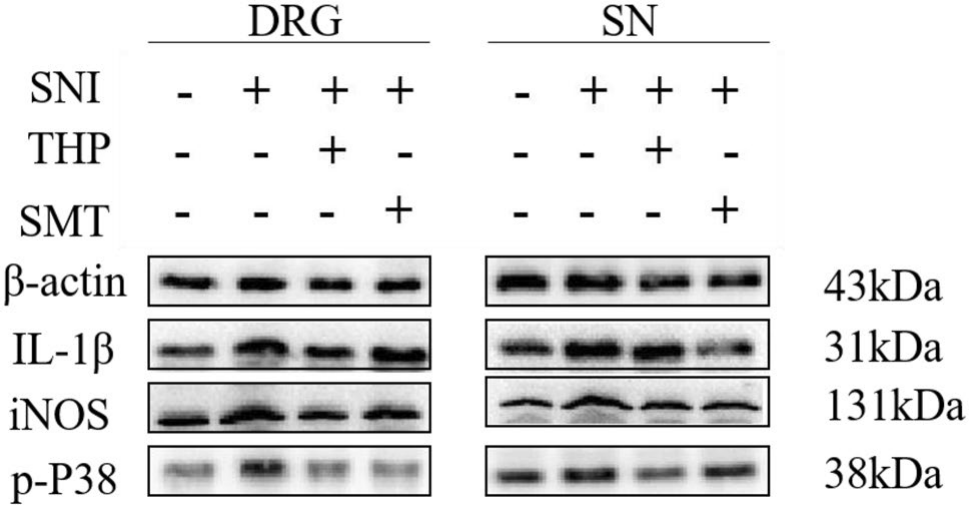
Effects of THP administration on the expression of related proteins in the SNI model. Total proteins were extracted from DRGs and SNs in different groups. A western blot analysis was performed to measure p-P38, iNOS, and IL-1β protein levels (*n* = 3 per group). β-actin was used as a loading controls, and images acquisition were performed by Image Lab™ software.

In SNI model, distal SNs and DRGs were separately collected for Western blotting. We determined the iNOS, p-P38, and IL-1β protein levels in SNs and DRGs. iNOS, p-P38, and IL-1β protein expression increased in the SNI group compared with the Sham group, while THP or SMT significantly decreased iNOS, p-P38, and IL-1β expression (Fig. 6).

### Effects of THP on the Signaling Pathways in Cellular Models

**Fig. 7:**
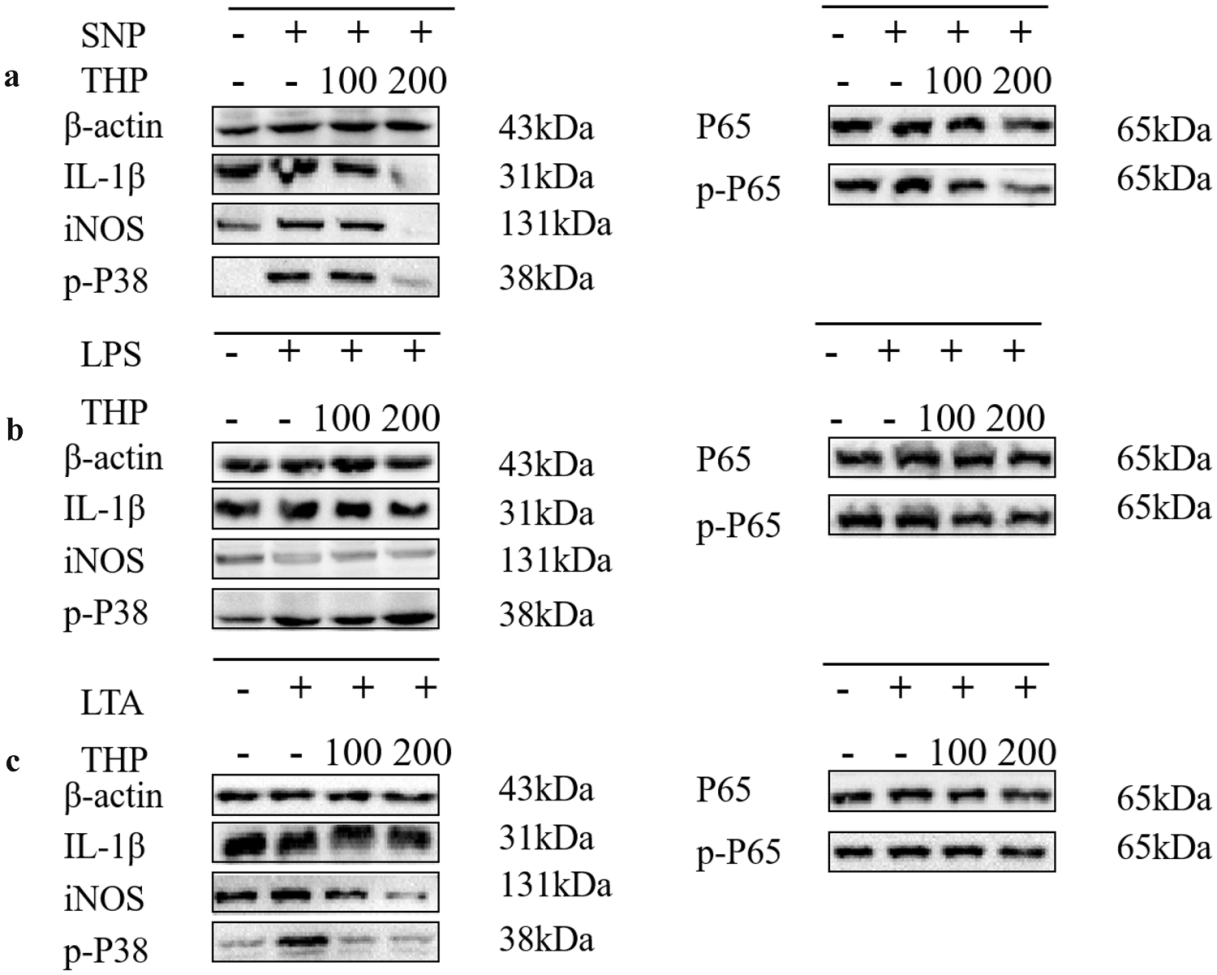
The expression of related proteins in the in vitro cell-induced model. A western blot analysis was performed to measure p-P38, iNOS, IL-1β, NF-κB, and p-NF-κB expression in different cell-induced models. β-actin was used as a loading controls, and images acquisition were performed by Image Lab™ software.

To explore the molecular mechanism of THP in the signaling pathway, the NF-κB and p38 MAPK pathways were investigated via Western blotting analysis. As shown in Fig. 7, SNP-induced N2a cells, LPS-induced BV2 cells, and LTA-induced astrocyte cells model increased the levels of p-P38, iNOS, IL-1β, and p-NF-κB. THP remarkably downregulated the increased expression levels of p-P38, iNOS, IL-1β, and p-NF-κB. The NF-κB total protein level did not significantly change in each group, but the phosphorylated levels significantly changed in different groups (Fig. 7a–c).

### Immunofluorescence Staining

**Fig. 8:**
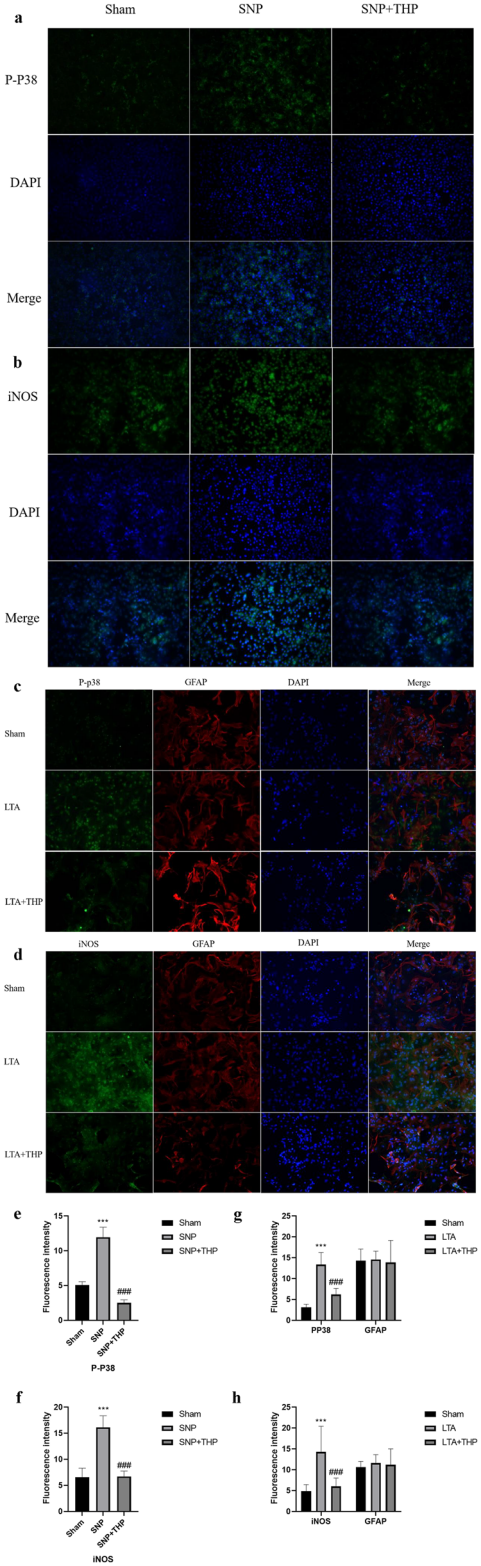
Immunofluorescence staining was used to examine p-P38, iNOS, and GFAP expression in different cells models. GFAP was used as a marker protein for astrocyte cells stained in the LTA induced cells model. No differences were observed in the expression of P38. Quantitative analysis was performed by ImageJ. Data are expressed as mean ± S.D. (***) *P* < 0.001 indicate significant differences compared with the Sham group; (###) *P* < 0.001 indicate significant differences compared with the SNP or LAT group.

As a complementary approach, SNP induced in N2a cells, while LTA induced astrocyte cells, and immunofluorescence was performed with p-P38, iNOS, and GFAP antibodies. Consistent with our findings above, THP significantly suppressed p-P38 and iNOS levels in different cells models (Fig. 8a–d). Quantitative analysis of the immunofluorescent colocalization has shown in Fig. 8 (Fig. 8e–i).

## DISCUSSION

Previous studies demonstrated that there are numerous mRNAs that are related to the regulation of neuropathic pain [24]. In this study, we used an mRNA microarray to analyze the remarkable differentially expressed mRNAs between the SNI and experimental groups. Herein, we used a standardized SNI-induced model that offered behavioral and molecular changes that closely mimic many features of clinical neuropathic pain. Through the mRNA microarray screening, the MAPK signaling pathway was selected as one of the biomarkers for regulation of neuropathic pain and neuroinflammation.

MAPK is activated in neurons and glia and contributes to inflammatory and neuropathic pain [25]. The MAPK family consists of three major members: ERK, p38, and JNK, which represents three different signaling cascades. p38 MAPK responds to external stress signals that are mainly activated by a number of inflammatory stimuli [26]. In the present study, our results demonstrated that the inhibition of MAPK suppresses the activation of NF-κB and ameliorated neuropathic pain behaviors. The previous studies showed that p38 MAPK is a key signaling pathway in activating neuroinflammation responses and contributes to neuropathic pain.

To clarify and specify the molecular mechanism of THP in neuropathic pain, the MAPK and NF-κB signaling pathways were subsequently investigated. Based on the previous remarkable anti-nociceptive mechanism of THP in aches and pains, the effects of THP on NF-κB- and MAPK- regulated proteins were evaluated. Our results demonstrated that THP exhibited significant effects on the phosphorylation of p38 and NF-κB activations. Furthermore, MAPKs are essential upstream regulators of transcription factor activities, and their signaling is critical for the transduction of extracellular oxidative stress stimuli into intracellular events. NF-κB and MAPK activations contribute to increased hyperalgesia via the increased production of various cytokines [19].

In recent years, researchers have shown that NF-κB, a transcription factor involved in the genesis and amplification of inflammatory insults at various tissue sites, can be activated by oxidative stress and cause the formation of p-NF-κB. And after phosphorylation, NF-κB could translocate to the nucleus and bind with the corresponding sequence of the target gene such as pro-inflammatory factors and then upregulates the expression of IL-1β [27]. Many experimental studies in recent years have provided evidence that inflammatory mediators induce or increase neuropathic pain, the key step of which is the activation of NF-κB pathway. Recently, evidence has emerged that there is increased expression of IL-1β in varieties of neuropathic pain models such as chronic sciatic nerve injury and SNI-induced peripheral neuropathic pain. Previous studies reported that inflammation and nitrosative stress are responsible for the pathophysiological alternations of neuropathic pain [11]. Moreover, in agreement with the previous evidences, THP remarkably inhibited the activation of NF-κB and also decreased the levels of pro-inflammatory factors IL-1β. These results prove our hypothesis, which is that THP alleviates SNI-induced neuropathic pain by suppressing nitrosative stress, inhibiting the activation of NF-κB, downregulating the expression of inflammatory factors, and mitigating neuroinflammation.

SNP (Na2 [Fe(CN)5NO]) is widely used in clinical and pharmacological studies as a NO donor. NO is a free radical that is physiologically produced through the L-arginine/NO synthase (NOS) pathway. Its anti-inflammatory effects and overproduction can initiate neurotoxic actions under pathological conditions, while it can also act as a neurotransmitter under physiological conditions [28, 29]. In this study, we designed an in vitro experimental model in SNP-induced N2a cells, LPS-induced BV2 cells, and LTA-induced astrocyte injury to understand neuropathic pain. The aim of the present study was to investigate the protective effects of THP on SNP-induced N2a neuronal cells, LPS-induced BV2 microglial cells, and LTA-induced astrocytes and its mechanism in the signaling pathway. Our results demonstrated that the induced neurotoxic effect was protected by THP. iNOS expression was induced in a different model. These results suggest that THP may prevent neuronal damages via the prevention of NO-mediated toxicity.

Mounting evidence has shown that iNOS is involved in the development of neurodegenerative diseases such as pain and multiple sclerosis (MS). iNOS is most intimately associated with inflammation and pain among all isoforms [30]. NO is generated from iNOS, which is important in the pathophysiology of inflammatory and neuropathic pain, as supported by its expression in glial cells after peripheral nerve injury. Activated glia release NO and anti-/pro-inflammatory cytokines such as TNFɑ, IFNγ, and IL- 1β [31]. Therefore, in the present study, we hypothesized that THP may be implicated in neuropathic pain and acts by reducing nitrosative stress and inflammatory markers.

## SUMMARY

Our study identified THP as a potential treatment to alleviate SNI-induced peripheral neuropathic pain. THP was consistent in exhibiting its analgesic properties toward mechanical hyperalgesia and cold allodynia, when THP treatment was provided throughout the development and progression of neuropathic pain. This outcome is due to the anti-inflammatory property exerted by THP. We demonstrated that THP successfully reversed the production of NO in blood plasma as well as in SN and DRG tissues. These data indicate that THP can ameliorate the neuropathic pain, along with inhibiting of iNOS and NO production. Therefore, there is great potential to use THP as a drug for the management of pain in neuropathic conditions through suppression of the inflammatory events that occurs due to nerve injury. THP can alleviate SNI-induced peripheral neuropathic pain, and the mechanism might be the inhibition of nitrosative stress-mediated activation of NF-κB, downregulation of the expression of inflammatory factors, and mitigation of neuroinflammation. Further studies are needed to clarify its deeper mechanism and clinical utilization.

## Data Availability

The raw date and materials about this article are available from the corresponding author.

## Abbreviations

SN: Sciatic nerve
SNI: Spared nerve injury
THP: Tetrahydropalmatine
MWT: Mechanical withdrawal threshold
DRG: Dorsal root ganglion
